# A dynamic interplay between membranes and the cytoskeleton critical for cell development and signaling

**DOI:** 10.3389/fpls.2014.00335

**Published:** 2014-07-16

**Authors:** Clément Thomas, Christopher J. Staiger

**Affiliations:** ^1^Laboratory of Cellular and Molecular Oncology, Department of Oncology, Public Research Centre for HealthLuxembourg, Luxembourg; ^2^Department of Biological Sciences, Purdue UniversityWest Lafayette, IN, USA

**Keywords:** actin, cytoskeleton, membrane trafficking, microtubules, signaling lipids

Diverse cellular processes that underlie plant development and response to environmental cues rely on a dynamic interplay between membranes and the cytoskeleton. The original research and review articles assembled in this Research Topic provide a valuable insight into our current understanding of how actin filaments and microtubules physically and functionally interact with membranes and cell wall and *vice versa*.

## Actin-binding proteins: old and new

Actin-binding proteins come in hundreds of different varieties and are the governors for cellular cytoskeletal organization and turnover. The rate limiting step for generating actin filaments from a monomer pool *in vitro*, and presumably *in vivo*, is formation of a seed or nucleus of three actin subunits. Formins are conserved actin nucleation factors which, in plants, take on special relevance for the presence of secretion signals, transmembrane domains and lipid interaction motifs that permit their function on or near cellular membranes. In an up-to-date review, Cvrčková (2013) discusses the myriad ways that plant formins are thought to associate with membranes and their function(s) at various subcellular locations, especially the interface between cortical actin cytoskeleton–plasma membrane–cell wall.

The monomer-binding protein, profilin, was the first conventional plant actin-binding protein to be identified more than two decades ago. Sun et al. (2013) briefly review the long history of discovery research surrounding this abundant regulator of actin dynamcis. Biochemically, isoforms from maize and Arabidopsis have been well characterized and these bind three cellular ligands: monomer of G-actin, proteins with contiguous stretches of proline residues, and phosphoinositide lipids. More recently, it has been discovered that plant profilins are localized or function at cellular membranes and organelles via their interaction with nucleators like the formins; here, profilin-actin complexes likely provide a supply of subunits to polymerize new actin filaments. Whether this actin assembly powers vesicle or organelle movements, as in the case of mammalian endosomes or pathogenic intracellular bacteria, is an area for future investigations.

One striking difference between the repertoire of actin-binding proteins from plants and animals is that many types of actin-membrane adaptors present in animals are absent from plants. Plants in turn own a plant-specific superfamily of actin-binding proteins, called the Networked (NET) proteins; presumably these are able to connect actin filaments to various membrane compartments. Here, Hawkins et al. (2014) report a phylogenetic analysis including NET gene sequences across a large range of species. Their data support that the NET superfamily emerged early in the evolution of vascular plants and has continuously developed and diversified coincidentally with the complexity of plant species.

## Cortical microtubules and cell wall assembly

A remarkable parallelism between membrane-associated microtubules in the cortical cytoplasm and cellulose microfibrils in the cell wall has captured the imagination of plant biologists for a half century. A review by Lei et al. (2014) synthesizes a broad range of research on this topic, from thoughts on how the primary wall regulates cell morphogenesis to the nature and dynamic visualization of the cellulose synthesis machine (CESA complex) that resides in the plasma membrane, and to how cortical microtubules guide or influence the trajectory of CESA movement in the plane of the membrane. Most importantly, these experts highlight the recent discovery and characterization of microtubule–CESA linkers, known as CSIs.

During secondary cell wall assembly, massive localized cellulose depositions in the cell wall are predicted and patterned by bands of cortical microtubules interspersed by regions devoid of microtubules. In elegant work by Oda and Fukuda (2013), a mutually inhibitory interaction beween cortical microtubule bundles and membrane domains is elucidated. They review the foundational work that reveals the switching on and off of ROP GTPases by GAP and GEF proteins that generate membrane domains. An effector of the activated ROP11, MIDD/ICR/RIP, regulates microtubule depolymerization via the kinesin-13A family member at these membrane domains.

## Trafficking on the cytoskeleton toward the plasma membrane and the nucleus

Cytoskeleton-dependent trafficking of cell wall and membrane components is crucial for establishing and maintaining polar growth. Dissecting the underlying, highly coordinated, regulatory pathways remains a major challenge. In this context, mutations in genes encoding the unconventional plant kinesin ARK1 or the ARF-GTPase modulator AGD1 were reported to induce strikingly similar root hair developmental defects and cytoskeletal aberrations, suggesting that ARK1 and AGD1 specify root hair tip growth via common molecular pathways. To further examine this possibility, Yoo and Blancaflor (2013) compared root hair defects in various double mutant combinations to *ark1* and *agd1*. Furthermore, they analyzed the distribution of the small GTPases ROP2 and RABA4b in both *ark1* and *agd1* root hairs. Their data confirm that ARK1 and AGD1 functionally overlap in maintaining the stability of small GTPases involved in root hair tip growth. They also point to divergences with ARK1 having a broad function during the entire root hair developmental program and AGD1 being more specifically involved in the early stages of root hair initiation and tip growth.

Batzenschlager et al. (2013) bring us into a functional study suggesting that the *Arabidopsis* γ-tubulin complex (γ-TuC) components GCP3-interacting protein1 (GIP1) and GIP2 are key determinants of nuclear shaping and nuclear envelope organization. The authors also report the identification of a novel and direct partner of AtGIP1, namely AtTSA1, which partially co-localizes with AtGIP1 at the nuclear envelope. Based on AtTSA1 domain signatures and previously identified partners, they suggest a model in which AtGIP1-AtTSA1 interaction is involved in the anchoring of γ-tubulin complexes at the outer nuclear envelope as well as in chromatin regulation within the nuclear compartment, positioning GIPs as a key regulator of a nucleo-cytoplasmic continuum.

## Lipid regulation of the cytoskeleton and the exocyst complex

Protein–lipid interactions dominate signal transduction pathways, but also influence cortical cytoskeletal organization, and *vice versa*. In their article, Pleskot et al. (2014) summarize current knowledge about the regulation of actin dynamics by membrane phospholipid signaling molecules, with a particular focus on phosphatidylinositol 4,5-bisphosphate (PIP_2_) and phosphatidic acid (PA). Following a brief description of the structural properties of PIP_2_ and PA that are relevant to their functions at the plasma membrane, the authors discuss the interrelationships between PIP_2_ and PA biosynthesis and the actin cytoskeleton. In addition, they review how PIP_2_ and PA induce cytoskeletal changes via the regulation of specific actin-binding proteins, e.g., capping protein, or ROP small GTPases. Finally, the authors explain why they anticipate a synergistic regulation of cytoskeletal regulators by various phospholipids and an intense crosstalk between PIP_2_ and PA signaling to the actin cytoskeleton.

The exocyst is a multiprotein complex, conserved across kingdoms, that tethers secretory vesicles to the plasma membrane during polarized growth. Synek et al. (2014) review linkages between this complex and the cortical actin and microtubule cytoskeletons, as well as small G-proteins. They further portray the exocyst as a key hub for coordinating exocytosis and signal transduction/sensing of cell wall stresses. Although most of this article summarizes influential studies and fundamental data from yeasts and mammalian cells, the path forward for plant biologists is clearly defined.

### Conflict of interest statement

The authors declare that the research was conducted in the absence of any commercial or financial relationships that could be construed as a potential conflict of interest.
